# Ontology-Based Method for Fault Diagnosis of Loaders

**DOI:** 10.3390/s18030729

**Published:** 2018-02-28

**Authors:** Feixiang Xu, Xinhui Liu, Wei Chen, Chen Zhou, Bingwei Cao

**Affiliations:** School of Mechanical Science and Engineering, Jilin University, Changchun 130022, China; xufeixiangsdut@163.com (F.X.); liuxh@jlu.edu.cn (X.L.); zhouchen17@mails.jlu.edu.cn (C.Z.); caobw16@mails.jlu.edu.cn (B.C.)

**Keywords:** loaders, fault diagnosis, ontology, CBR, RBR

## Abstract

This paper proposes an ontology-based fault diagnosis method which overcomes the difficulty of understanding complex fault diagnosis knowledge of loaders and offers a universal approach for fault diagnosis of all loaders. This method contains the following components: (1) An ontology-based fault diagnosis model is proposed to achieve the integrating, sharing and reusing of fault diagnosis knowledge for loaders; (2) combined with ontology, CBR (case-based reasoning) is introduced to realize effective and accurate fault diagnoses following four steps (feature selection, case-retrieval, case-matching and case-updating); and (3) in order to cover the shortages of the CBR method due to the lack of concerned cases, ontology based RBR (rule-based reasoning) is put forward through building SWRL (Semantic Web Rule Language) rules. An application program is also developed to implement the above methods to assist in finding the fault causes, fault locations and maintenance measures of loaders. In addition, the program is validated through analyzing a case study.

## 1. Introduction

With the development of industry and technology, the modern engineering mechanisms, e.g., loaders, which are becoming increasingly complex in structural systems, hydraulic systems and electrical systems, have higher failure rates. Moreover, because of bad working circumstances, such as forest areas with slippery terrain, hilly areas and mine areas with rough terrain, etc., loaders have to confront different kinds of faults frequently, which make it difficult to find faults and diagnose faults in time [1]. In this context, it is urgent to study the loaders fault diagnosis method. Over the past decade, many methods have been developed in the fault diagnosis domain, for example, methods based on signal analysis, information knowledge-based methods, and model-based methods [2]. In particular, the model based on fault diagnosis is nowadays accepted as a powerful tool to solve fault detection problems in technical processes [3].

The model-based fault diagnosis method was originally developed by Beard, in 1971, to replace hardware redundancy by analytical redundancy [4], which has been developed for linear or linearized models. For example, Steven X. Ding et al. [5] proposed a data-driven framework for the design of observer-based fault detection and control systems. The linear model represents the system behavior only near one operating point. Indeed, when faults occur, the operating point changes; therefore, a linear model is not representative [6]. Thus, to consider an operating range of nonlinear systems, the Takagi-Sugeno (T-S) fuzzy model has been applied in the diagnosis of complex nonlinear systems [7]. The fault estimation observer was developed in [3] for discrete-time T-S fuzzy systems based on piecewise Lyapunov functions. The unknown inputs of observers are designed in a finite frequency domain for T-S fuzzy fault detection [8]. T. Youssef et al. [6] used a Proportional Integral observer design for actuator and sensor faults estimations based on the T-S fuzzy model. Although the fault diagnosis methods mentioned above can diagnose loaders’ faults by using a small amount of real-time data, they require an explicit model representing the input–output relationship; the fault diagnosis performance of loaders relies on this model’s accuracy [9]. Moreover, using the above methods, it is hard to present a universal approach for the fault diagnosis of all loaders. Different kinds of loaders have different components and design principles, resulting in various diagnostic methods. Even for the same type of loader, components might be quite different due to various factors, such as repair, customization or update. To solve these problems, case-based reasoning (CBR) is exploited, as does not need an explicit and accurate model. CBR is an important method used in learning and solving problems in artificial intelligence [10], which obtains the solution of the target case by visiting a source case that is similar to the target case in its case library based on past practical experiences [11,12]. In this context, the faults of all loaders can be diagnosed based on past experiences. What is more, its problem-solving ability increases with the accumulation of experience. One disadvantage of CBR is that the fault diagnosis will be invalid when there is no corresponding case. Therefore, the rule-based reasoning (RBR) method is proposed for diagnosing loaders’ faults once CBR fails.

CBR and RBR fault diagnosis methods have the following shortcomings in the knowledge management of fault diagnosis.

(1) It is difficult for computers to understand and integrate fault diagnosis knowledge of loaders, especially when there are heterogeneous knowledge types, wide knowledge sources and different storage types of knowledge.
(2) It is difficult to reuse the existing loaders fault diagnosis model. Numerous scholars have to reestablish models and methods during the study of loaders fault diagnosis, resulting in the waste of research energy.

To overcome the two defects mentioned above, the concept of ontology was proposed, which is a mechanism that describes concepts and their systems’ relationships [13,14]. As the basis of semantic knowledge, ontology can make concepts, vocabularies, attributes and other knowledge of the domain semantically, which enables the computer to easily understand and integrate the knowledge, thus sharing and reusing knowledge [15,16]. Hence, in this paper, we will exploit the ontology concept in our method.

In this paper, we combine ontology, CBR and RBR to realize the fault diagnosis of loaders, which assists users in finding the fault causes, fault locations and maintenance measures of loaders.

The rest of this paper is organized as follows: Section 2 briefly introduces the background and related works; Section 3 explains the overall structure of the fault diagnosis system; Section 4 focuses on the construction of the fault diagnosis ontology model; Section 5 describes the ontology-based CBR fault diagnosis method; Section 6 describes the ontology-based RBR fault diagnosis method; Section 7 describes the system implementation and validation; The final section contains the study’s conclusions and suggestions for further work.

## 2. Background and Related Works

Previous research regarding loaders fault diagnosis methods mainly covers ontology and CBR/RBR methods. The following is the background and literature review of related works.

### 2.1. Ontology

Ontology has been applied in fault diagnosis, decision analysis, prediction and early warning in recent years. The ontology model is described as O = <C, OP, DP, I> [17,18], where C is the set of classes; OP is the set of object properties; DP is the set of data properties; and I is the set of instances.

OWL (Web Ontology Language) is an ontology description language, recommended by W3C (World Wide Web Consortium) international standards, which has a strong semantic ability to describe classes and attributes [19]. OWL1 is used to describe the ontology model in this paper, which consists of OWL Lite, OWL DL and OWL Full sublanguages. OWL DL is selected as the description language because it can incorporate description and reasoning. Sparql (Simple Protocol and RDF Query Language) is a language that enables query information in the OWL model [20]. The Jena inference engine can parse and reason ontology model. The combination of OWL, Jena inference engine and Sparql is the hot direction of knowledge representation [21]. Yu-Jun Wang et al. [22] described food cold chain quality ontology by using OWL and the Jena inference engine. The threshold of logistics parameters was queried, to obtain parameters’ statuses based on Sparql. Xiao-Ci Huang et al. [23,24] described a water quality monitoring ontology model based on OWL and used the Jena inference machine to analyze the ontology model. Then, Sparql was used to query the threshold of water quality related parameters, so as to get the statuses of parameters.

With the rapid development of knowledge technology, ontology has been applied in areas like fault diagnosis. Rong Chen et al. [25] put forward an ontology-based knowledge modeling approach for fault diagnosis for rotating machinery. The ontology model was built to integrate fault diagnosis knowledge of rotating machinery. Furthermore, rules were added to allow the reasoning of fault diagnosis knowledge. However, this research did not deduce root causes for faults but described the reasons for general fault causes. In addition, the defined rules were relatively simple. Haizai Peng et al. [26] proposed a fault diagnosis method for conveyor-based ontology which was used to construct a knowledge basis for conveyor fault diagnosis. This study established the mapping relationships between fault phenomenon ontology, fault cause ontology and fault solution ontology, which have given us the guidance to manage knowledge representation well and independently through constructing sub-ontologies. G. Medina-Oliva et al. [27] proposed a fleet-wide approach based on ontologies in order to capitalize on knowledge and data to help decision makers to identify the causes of abnormal operations. The ontology modelling process in G. Medina-Oliva’s research was similar to this paper, which used Protégé to construct ontology coded in OWL, and built Semantic Web Rule Language (SWRL) rules to diagnose faults. Alfonso Castro et al. [28] designed a multimedia service and resource management architecture for fault diagnosis. This architecture includes three types of automatic reasoning: heterogeneous, ontology-based and Bayesian reasoning. The combination of Bayesian reasoning and ontology-based reasoning performed better on incomplete datasets and in the presence of uncertainty, which could avoid the data loss problem caused by data acquisition devices in this paper. FD Samirmi et al. [29] aimed to develop an improved ontology model for transformer fault diagnosis by applying the fuzzy ontology. Similar to this paper, Protégé software and OWL DL were applied to build the ontology model. Based on ontology, fuzzy theory was introduced to solve uncertainty or imprecision problems for fault diagnosis.

### 2.2. CBR/RBR Method

In recent years, CBR and RBR have received more and more attention and have been applied for fault diagnosis as two main techniques in artificial intelligence and expert systems. Tomas, Gang Ma et al. [30] put forward an intelligent fault diagnosis method for power equipment by retrieving historical cases based on CBR. Olsson, T. et al. [31] classified the fault diagnoses and deduced the fault probabilities regarding CBR. Dong-yang Dou et al. [32] proposed an RBR method to achieve fault diagnosis for rotating machines. Xiao-Wen Deng et al. [33] designed an RBR based fault diagnosis expert system for wind turbines. Olsson, E. et al. [34] combined the acoustic signal and CBR to obtain the fault diagnosis of industrial robots. Dinh PhuocVo et al. [35] presented a hybrid knowledge-based system which proposed to a diagnosis approach for test engineers based on the combination of CBR and RBR to be used when an incident occurs.

However, the ability to understand knowledge semantically based on CBR and RBR is very poor [36,37]. As we all know, fault diagnosis based on CBR and RBR is usually a high intelligence activity, and experts often begin their diagnosis by observing, querying and interpreting faults diagnosis experience knowledge. In this way, experts form vocabularies of semantic expressions, but semantic ambiguity often occurs in experience observations and queries when experts do not know the exact expressions or the related concepts—though they may have some professional knowledge [36]. Hence, ontology is exploited to improve the ability to understand knowledge semantically based on CBR and RBR. Nadjette Dendani-Hadiby et al. [38,39] established an ontology model to obtain the semantics of knowledge, and then integrated CBR with ontology to develop a fault diagnosis system for steam turbines. An-mei Zhou et al. [40] combined an ontology model and the RBR method to obtain intelligent fault diagnosis for wind turbines, and the feasibility of the method was analyzed with practical cases. Based on an ontology model, Xiao-ci Huang et al. [41] designed custom rules to determine intelligent fault diagnoses for off-line Electronic Control Unit (ECU). Tung et al. [42] developed a solution retrieval system based on ontology, CBR and RBR. The experimental results show that this method can improve the accuracy of retrieval cases and reduce retrieval time prominently. Shao-li Chen et al. [37] built the disassembly knowledge model based on ontology, and then used CBR and RBR to obtain automated decision-making for the disassembly of mechanical products. Combining ontology and RBR, Zhou Qiang et al. [43] presented an intelligent fault diagnosis method for machine tools, and validation occurred by studying cases. D. Wang et al. [44] presented a new approach to the fault diagnosis of power transformers based on ontology and RBR. Based on the ontology and fault tree analysis methods, Wu Chun-yin [45] established a fault diagnosis knowledge model, and the CBR method was proposed to diagnose the faults of a tractor.

Inspired by previous work, as a novel attempt, ontology, CBR and RBR are applied into the field of loaders fault diagnosis in this paper.

## 3. The Overall Structure of Fault Diagnosis System

**Figure 1:**
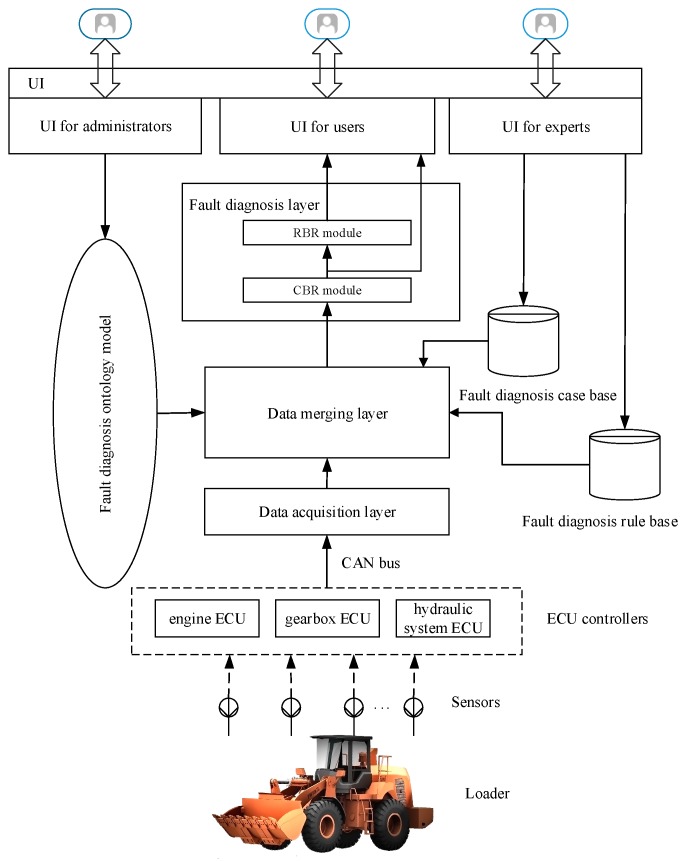
Overall structure of loaders fault diagnosis system. CBR, case-based reasoning; RBR, rule-based reasoning; UI, user interface; CAN, Controller Area Network; ECU, Electronic Control Unit.

The main purpose of this paper is to design a loaders fault diagnosis system to help users find the fault causes, fault locations and fault maintenance measures of loaders in adequate time, based on ontology, CBR and RBR. Figure 1 shows the overall framework of the fault diagnosis system. The running parameters of the loaders are integrated in the data merging layer through data acquisition and transmission, based on sensor and embedded technologies, and then running parameters, the ontology model, the fault diagnosis case base and the fault diagnosis rule base are gathered in the data merging layer. Following this, fault diagnosis is achieved in combination with CBR and RBR; finally, fault diagnosis results are displayed on a user interface. As can be seen from Figure 1, the data collected by the sensor is the database of the whole fault diagnosis system. On one hand, in the CBR module, the collected data is used as one feature index of case-retrieval to match a suitable case in the case base. On the other hand, in the RBR module, the collected data is compared with data threshold values to judge instances of the *Parameters* class of ontology model, and then fault diagnosis is realized by combining ontology and defined rules. The function of each layer is described in detail.

- Data acquisition layer. Firstly, the sensors collect data (such as engine speed, oil pressure of engine, hydraulic oil temperature etc.) every 2 s. Secondly, the collected data is encapsulated by three ECU controllers, including engine ECU, gearbox ECU and hydraulic system ECU. Thirdly, the data acquisition device gathers and processes all data from the three ECU controllers according to the Controller Area Network (CAN) protocol every five minutes. On one hand, within 5 min, the data acquisition device processes 150 data from each parameter to obtain an average value for each parameter. In this way, the average values of running parameters for fault diagnosis are more useful and effective. On the other hand, data is transferred to the data merging layer every 5 min, with the aim of reducing the frequency of data transmission so as to decrease the data processing pressure on the data acquisition device and data merging layer. This data acquisition device is developed by embedded technology, and it is composed of an Microcontroller Unit (MCU), a power module, an Liquid Crystal Display (LCD) module, a CAN module and a 3G module. MCU selects the stm32f103 microprocessor as the main control unit, which is responsible for receiving data, analyzing data and sending data. The power module mainly provides the power supply for the data acquisition device to ensure normal operation. The LCD module is used to display the operating data. The CAN module is used to receive data from three ECU controllers. The 3G module is responsible for sending data to the data merging layer. At last, the processed data is transferred to the data merging layer via the Internet in the form of Transmission Control Protocol/Internet Protocol (TCP/IP). The collected data lays the data foundation for the fault diagnosis layer to diagnose loaders’ faults. The data package from each ECU controller has 29 bytes, and then the data merging layer transferred from the data acquisition device has 29 × 3 = 87 bytes. Given that the loader works 8 h per day, and the historical data can be saved for three months, the memory size for collected data is 87 × 8 × 60/5 × 30 = 250,560 bytes = 245 MB. Currently, there is one diagnosed loader; the memory size for collected data is 245 MB. Supposing that the ontology-based fault diagnosis system will diagnose 1000 loaders in the future, then the memory size for collected data is 245 MB × 1000/1024 = 239 GB.
- Data merging layer. The data merging layer is used to integrate collected data, the ontology model, the fault diagnosis case base, and the fault diagnosis rule base. The data collected by sensors is transferred to the data merging layer through the data acquisition device. The fault diagnosis ontology model semantically handles the relevant information of loaders fault diagnoses, so that the information can be stored and expressed. The case base summarizes previous diagnosis experience and provides case support for CBR. The rule base, which is defined by experts and expressed in the form of SWRL, provides rule support for RBR. The data merging layer is responsible for laying the foundation for the fault diagnosis layer, which is of great significance. Moreover, the ontology model, rule base and case base can be updated and improved continually. With the increase in data in the data merging layer, it will be able to diagnose more faults and can diagnose more accurately.
- Fault diagnosis layer. This layer is the core part for diagnosing loaders’ faults. Based on the fault diagnosis ontology model, the CBR module is used for fault diagnosis following four steps: feature selection, case-retrieval, case-matching and case-updating. The CBR module is able to obtain accurate fault diagnoses because it exploits historical cases which have actually happened. Once the CBR module fails, due to the lack of corresponding cases, the RBR module will be executed. The RBR module is used to deduce fault causes, fault locations and maintenance measures based on defined SWRL rules which make up the fault diagnosis rule base. By making full use of the advantages of the ontology model in knowledge management, CBR and RBR can obtain accurate and effective fault diagnoses of loaders.
- UI (user interface). The UI was mainly developed for users, experts and administrators. Users (including drivers, operators and manufacturers) can obtain fault diagnosis results of loaders through UI. Experts are responsible for constructing and managing the case library and the rule library. Administrators mainly design and supervise the fault diagnosis ontology model. The results, including fault causes, fault locations and maintenance measures, can be obtained through UI. The UI application program was developed with the Visual Studio2017 software platform and C# language under the Windows 7 system.

## 4. Construction of the Fault Diagnosis Ontology Model

This section mainly introduces the fault diagnosis ontology model including ontology parsing and querying to lay the foundation for CBR/RBR.

### 4.1. Construction of the Ontology Model

**Figure 2:**
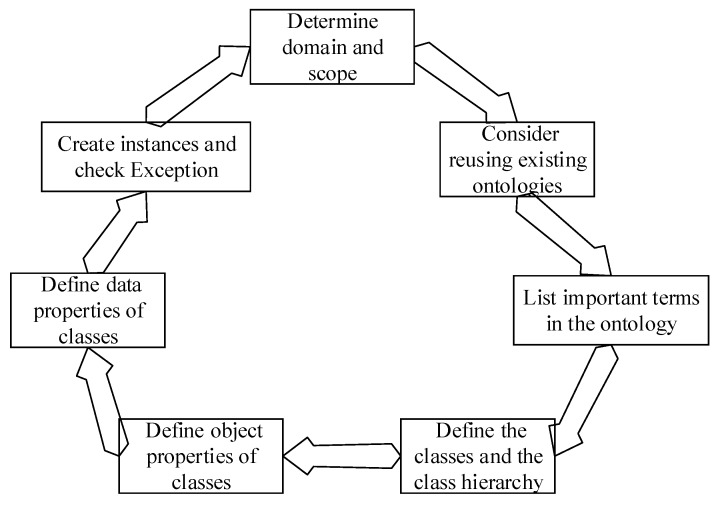
Seven-Step Method for ontology modeling.

Protégé [46] software was selected for building the fault diagnosis ontology model because of its user-friendly interface, powerful tools and data checking feature [47]. The ontology proposed in this paper is mainly used in the field of loaders fault diagnosis; thus, it can be classified as a highly specialized domain ontology. Currently, there are many kinds of approaches for constructing domain ontologies, i.e., METHONTOLOGY, SENSUS, TOVE, IDEF5 and the Seven-Step Method. Toshihiro Uchibayashi et al. [48] proposed a domain specific sub-ontology derivation end-user tool for the semantic grid, where ontology is built through extracting the sub-ontologies. This method is suitable for large-scale ontology domains and will be applied in this paper, when fault diagnosis ontology increases gradually. Hu qingxi et al. [49] built an ontology for representing the production process knowledge of workshops based on the IDEF5 method which has disadvantages in technical support. Zhou Yong [50] constructed an improved ontology model based on machine learning (BP neural network) which changed the mode of multi strategy merging in ontology mapping. S. Chaware et al. [51] integrated METHONTOLOGY and SENSUS to construct an ontology model for the shopping mall domain, where ontology can be prepared to get more correct information at a faster rate. However, this integrated method does not consider the collaborative and distributed construction of ontologies. The Seven-Step Method is among the top choices for building a domain ontology [22]. In addition, it not only enjoys detailed technical support and advantages in model creation, but also has advantages in the detailed modelling process [22]. Hence, this paper chose the Seven-Step Method, developed by the Medical Information Center of Stanford University [52], to construct the ontology model in the fault diagnosis domain, and it is shown in Figure 2. Next, the ontology modeling steps of the Seven-Step Method are described in detail.

• Determine the domain and scope

The research described in this paper is in the field of loaders fault diagnosis.

• Consider the reuse of existing ontologies

There is no suitable ontology that can be reused directly.

• List important terms in the ontology

The keywords of loaders fault diagnosis are engine, gearbox, oil temperature, gear pump, main valve, etc.

• Define classes and class hierarchy

The components of the loaders can be divided into four levels, from top to bottom, which are the device level, system level, assembly level, and part level. When the loader fails, a single fault can be aroused by various fault causes rather than one fault cause. Faults in low level components affect not only faults at same level, but also faults in higher levels. Thus, the causes of loaders’ faults are very complex.

**Figure 3:**
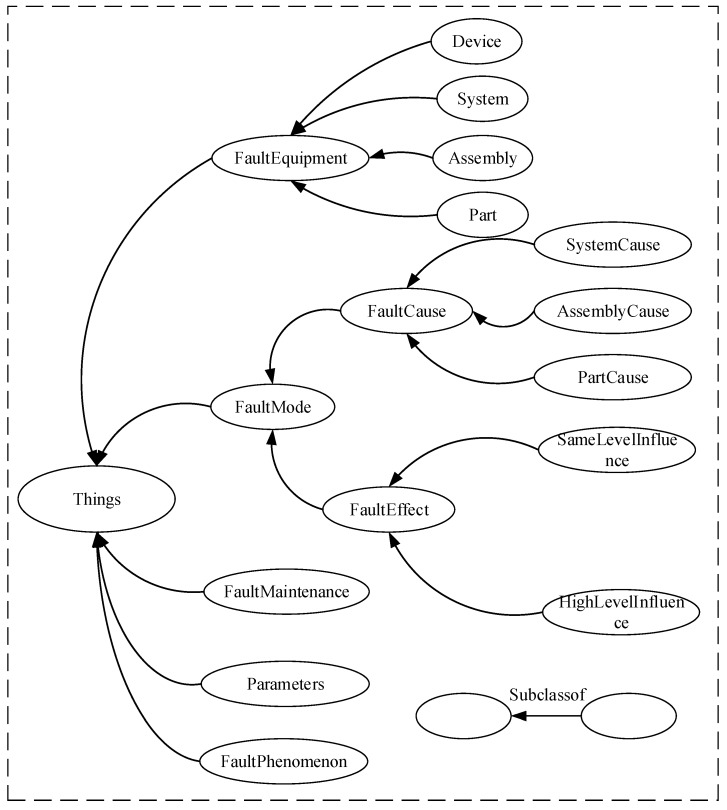
Classes in the fault diagnosis ontology model.

In order to handle the complexity of faults, the fault diagnosis ontology model has five defined classes: *FaultMode*, *FaultEquipment*, *FaultMaintenance*, *Parameters* and *FaultPhenomenon*, as shown in Figure 3. The *FaultMode* is composed of two subclasses: *FaultCause* and *FaultEffect* [40]. The *FaultEquipment* indicates the location of faults [43]. The *parameters* class expresses running data collected by sensors. The *FaultPhenomenon* indicates the phenomena when a failure occurs. The *FaultMaintenance* means that faults can be repaired by some measure.

• Define object properties of classes

As shown in Table 1, each object property has its corresponding domains and ranges. If an object property has an inverse property, then the inverse property has inverse domains and ranges.

(1) Property 1 indicates the change in operating parameters caused by the fault mode.
(2) Property 2 shows the relationship between the fault mode and the fault causes.
(3) Property 3 indicates the relationship between the fault components and their sub-components.
(4) Property 4 shows the relationship between the fault mode and fault effects. Property 5 and 6 are sub-properties of property 4, and property 5 indicates the relationship fault mode and its same level effects, and property 6 indicates the relationship fault mode and its higher level effects.
(5) Property 7 indicates that fault locations provide information support for maintenance methods.
(6) Property 8 indicates that the fault mode is able to be repaired through using maintenance methods.
(7) Property 9 shows that the fault mode happens in components of loaders.
(8) Property 10 indicates that the fault mode is accompanied by phenomena.

• Define data properties of classes

As shown in Table 2, each data property, similar to the object properties, also has its own domains and ranges.

**Figure 4:**
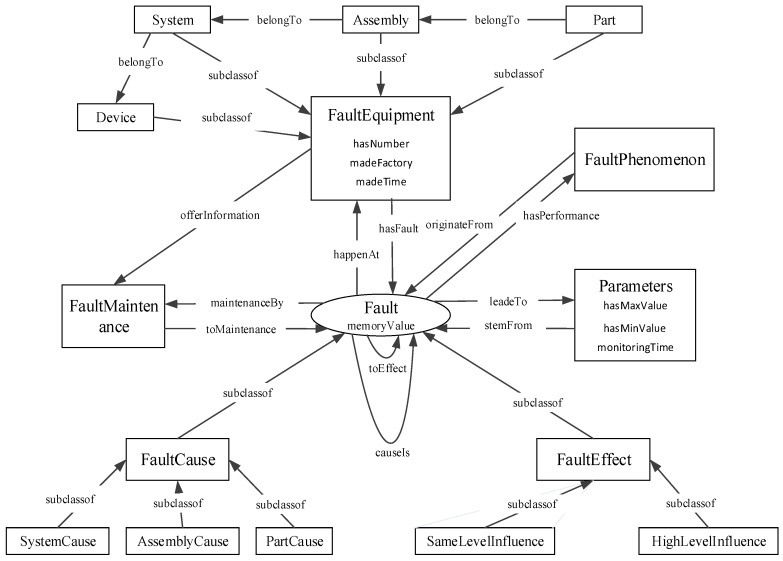
Structure of the fault diagnosis ontology model.

After the former six steps, the structure of the fault diagnosis ontology model is built, as shown in Figure 4.

• Create instances and check exceptions

Instances of fault diagnosis ontology model are divided into two parts. For the first part, instances of the parameters’ classes and their related properties are filled up by data from the loader’s data acquisition device. For the second part, system administrators and experts are responsible for instantiating other classes and properties according to the actual condition of the loaders. So far, the loaders fault diagnosis ontology model has been established after the instantiation. A pellet is used for checking exceptions, to verify the correctness of ontology model [22].

### 4.2. Parsing and Querying of the Ontology Model

After the fault diagnosis ontology model was constructed by Protégé software and described by OWL language, it needed to be parsed and queried to apply ontology model into the CBR/RBR method. As introduced in the second section, the Jena inference engine was used to parsethe ontology model in this paper, and the SELECT mode of the Sparql was selected to query the properties of the ontology model.

## 5. CBR for Loaders Fault Diagnosis

CBR, proposed by Schank (1983) [53], can simulates human cognitive processes and integrates empirical knowledge of different fields into a unified format [54]. CBR can achieve effective and accurate fault diagnosis of loaders. This is because that CBR can obtain causes of failure, fault locations and maintenance methods, as long as cases are able to be matched successfully in the case library. On the other hand, CBR can exploit historical cases which have actually happened and have been solved, to guarantee accuracy.

**Figure 5:**
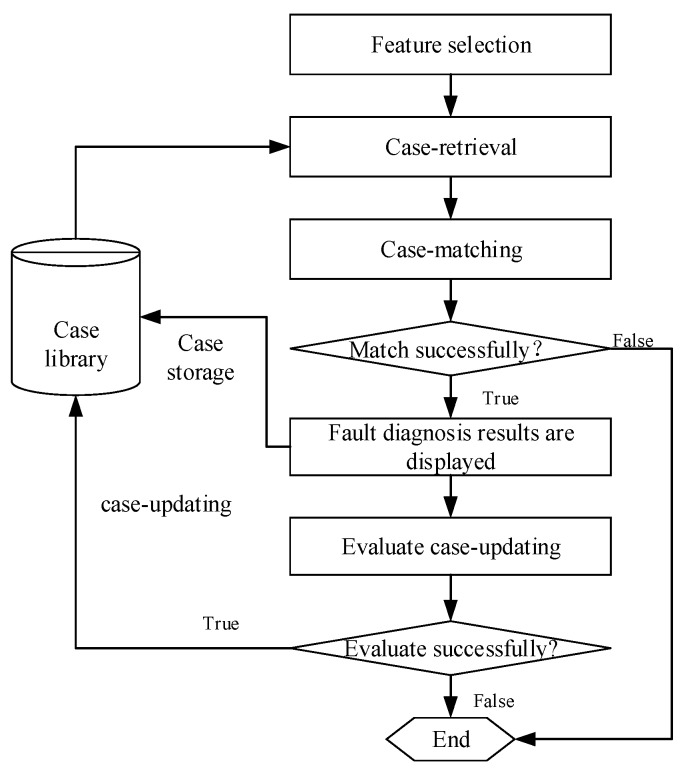
Process of the CBR based loaders fault diagnosis.

The process of CBR based loaders fault diagnosis is shown in Figure 5. Loader type, fault phenomena and parameters are selected as the feature indexes for calculating case similarities in the case library. When the similarity value is greater than or equal to the set value, it indicates that the case-matching has been successful, and the fault diagnosis results of the matched case are displayed directly; Then case-updating is evaluated, and the case library will be updated if the evaluation is satisfactory, or else the CBR method will end. When the similarity value is less than the set value, the CBR based fault diagnosis will fail and the diagnosis process will end. The fault diagnostic process is described in detail below.

### 5.1. Feature Selection and Case-Retrieval

Although there are a large number of loader types, repair methods are often similar for similar faults of the same loader type; therefore, the loader type is selected as an essential feature index. The maintenance methods used in the same fault phenomena can also be used as guidance to the maintenance person; thus, the fault phenomena index is another alternative. In addition, parameters obtained directly from the data acquisition device will change when faults occur, therefore, meeting the needs of the feature index. Hence, the three feature indexes are loader type, fault phenomena and operating parameters.

**Figure 6:**
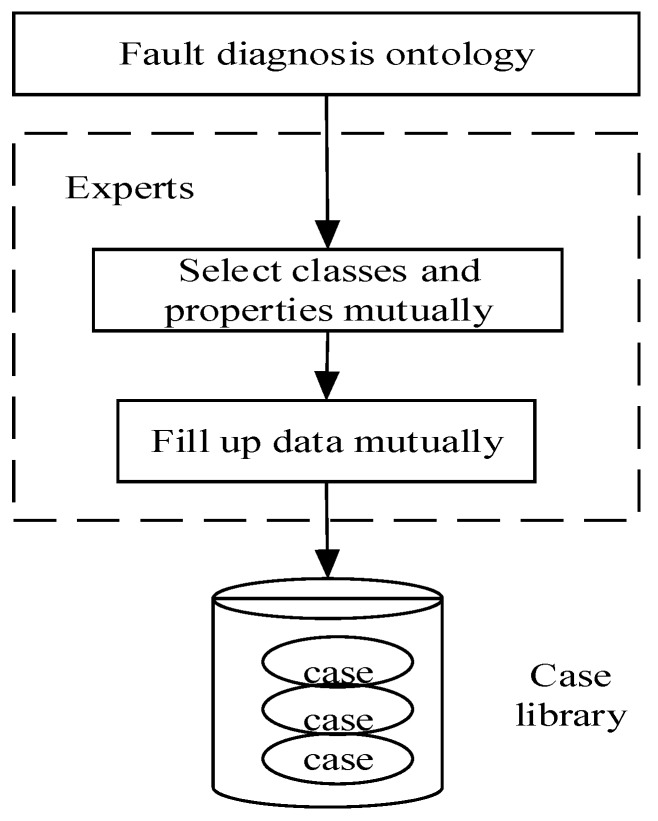
Process of constructing case library.

Case-retrieval refers to searching the fault diagnosis case library based on the above three feature indexes. Hence, the construction of the case library is of great significance. As shown in Figure 6, experts select the classes and properties of fault diagnosis ontology manually, and the corresponding data is filed manually regarding historical cases and events, and then fault diagnosis cases are built and stored in the case library. At this point, the case library is constructed. Moreover, the established ontology model with its semantic description can be retrieved with cases that are also built semantically in the above process of constructing the case base.

### 5.2. Case-Matching

The established ontology model with its semantic description can be retrieved with cases which are also built semantically, as mentioned above. Hence, case matching can be realized by matching instances of *hasNumber*, *FaultPhenomenon*, *Parameters* corresponding to three feature indexes between ontology models and cases. Similarity values are used to judge the similarity between to-be-diagnosed faults and the cases in the case library. The nearest neighbor algorithm [55] was used to calculate the similarity value in this paper, and its formula is shown in Equation (1).

(1) $$Sim(C_{i})=\sum_{j=1}^{m}(w_{j}*Sim(C_{ij}))$$

where *I* is the sequence number of the case in the case library. $Sim(C_{i})$ is the similarity value between to-be-diagnosed faults and the *i*th case. *J* is the sequence number of the feature index. *m* represents the numbers of all feature indexes. $w_{j}$ is the weight of the *j*th feature index. $Sim(C_{ij})$ is the similarity value between the *j*th feature of the *i*th case and the to-be-diagnosed faults.

The threshold of $Sim(C_{i})$ is set as Sim(C), which means that the case-matching is invalid when $Sim(C_{i})$ < Sim(C); otherwise case-matching is successful. The value, $w_{j}$, is directly defined by system experts to simplify the designing of this value. $Sim(C_{ij})$ is determined as follows in this paper:

(1) If the loader type is the same as the case in the case library, then $Sim(C_{i1})$ equals 1, or else $Sim(C_{i1})$ equals 0.
(2) If fault phenomena are identical to a case in the case base, then $Sim(C_{i2})$ equals 1, or else $Sim(C_{i2})$ equals 0.
(3) Operating parameters collected by sensors are calculated with Equation (2).
  (2) $$Sim(C_{i3})=\sum_{j=1}^{N}(m_{k}*Sim(f_{k}))$$
  where $m_{k}$ represents the weight of the *j*th operating parameter, which is determined directly by system experts, *N* stands for the number of operating parameter, $Sim(f_{k})$ is calculated by Equation (3), which represents the *j*th operating parameter similarity value between to-be-diagnosed faults and the case, $p_{k}$ denotes the *k*th operating parameter value of to-be-diagnosed faults, and $l_{k}$ represents the *k*th operating parameter value of the case.
  (3) $$Sim(f_{k})=1-\frac{p_{k}-l_{k}}{p_{k}}$$

### 5.3. Case-Updating

When case-matching is achieved successfully, the actual fault diagnosis results can be saved into the case library. However, too many similar cases will waste the storage resource and decrease the efficiency of case-retrieval and case-matching. Therefore, it is necessary to develop case-updating strategy to eliminate redundancy in the case library. Derbinsky et al. [56] proposed a case update method which forgets outdated cases and reinforces the memory of frequent cases. Based on this principle, the memory value (*M*) of each case has the following relationship:

(4) $$M=\frac{\pi }{2}-\arctan\Delta T,\Delta T\geq 0$$

where *M* stands for the value of the *memoryValue* data property in the fault diagnosis ontology, Δ*T* indicates the existence time of the case in the case base, and its initial value is set as “0”.

When cases are successfully matched, one of these cases is randomly retained, and its Δ*T* is set as “0”; then, the other cases are discarded. If we set the threshold of *M* to *Mt*, then the case is outdated and will be abandoned when *M* < *Mt*. By controlling suitable values of *Mt*, we can ensure that the case base does not become too narrowed down, oversized or skewed. In this way, a suitable number of cases can be saved in the case library. Additionally, cases in the case base can also be improved by different experts at any time to improve the effectiveness of case management. *∆T* of the modified case will be reinitialized to “0”.

## 6. RBR for Loaders Fault Diagnosis

Based on the fault diagnosis ontology model, RBR was used to process the loaders fault diagnosis when the CBR method failed due to the lack of proper cases.

### 6.1. Fault Diagnosis Rules

The standard SWRL language is used to express the fault diagnosis rules. Based on the analysis of classes and properties in the fault diagnosis ontology model, as shown in Table 3, the constructed SWRL rules are represented in this paper, and these rules are the basis of the ontology-based RBR method. To understand SWRL easily, two basic elements of syntax are introduced, as follows [57]:

(1) C (?x): If x is an instance of the class C or the value of its data property, then C (?x) is established;
(2) P (?x, y): If x and y are associated with the property, P, then P (?x, y) is valid.

In order to simplify the discussion here, we will only explain the following representative rules.

- Rule 1: If fault mode x occurs with the occurrence of fault phenomena y, then the direct fault locations of fault mode x are z.
- Rule 2: If fault mode x’s change derives from parameter y, and x happens at device component a, and fault mode z affects x at the same level, then the cause of x is fault mode z, and x has fault causes at the system level.
- Rule 5: If fault mode x’s change derives from parameter y, and x happens at system component a, and fault mode z affects x at a higher level, then the cause of x is fault mode z, and x has fault causes at the system level.
- Rule 8: If fault mode x happens at equipment y, and the reasons for the components’ faults are x, then the fault locations are y.
- Rule 11: If the fault mode x happens at equipment y, and the fault locations provide the method for troubleshooting z, then failure maintenance z can repair fault mode x.

### 6.2. RBR for the Loaders Fault Diagnosis Process

**Figure 7:**
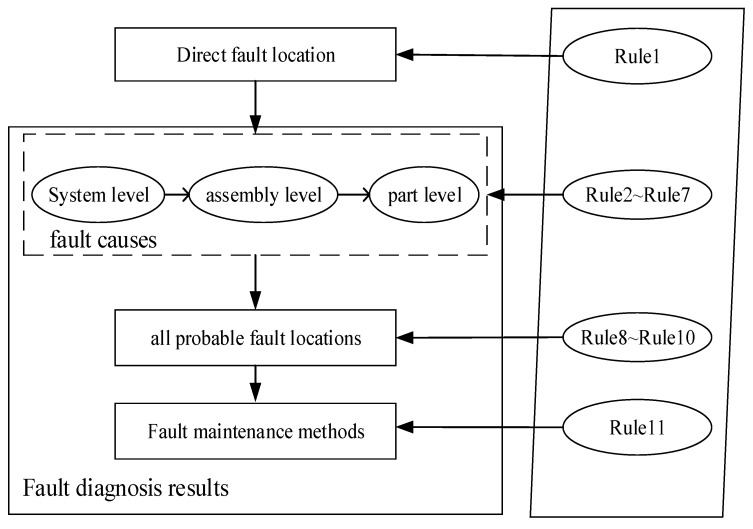
Process of loaders fault diagnosis based on RBR.

The process of loaders fault diagnosis based on RBR is shown in Figure 7. Firstly, the direct fault locations are deduced based on Rule 1 and the *FaultPhenomenon* property of fault diagnosis ontology. Secondly, Rules 2~7 are triggered to determine all probable fault causes by combining *stemFrom*, *happenAt* and *toEffect* from high to low levels. Rule 2 and Rule 5 are responsible for reasoning fault causes at the system level. Rule 3 and Rule 6 are triggered to determine fault causes at the assembly level. Rule 4 and Rule 7 are triggered to reason fault causes at the part level.

Thirdly, Rules 8~10 are triggered to deduce all possible fault locations based on the *happenAt* object property. Finally, in accordance with Rule 11, maintenance methods can be obtained by integrating the *offerInformation* and *happenAt* object properties.

Overall, combining fault diagnosis ontology and custom rules defined in Table 3, fault diagnosis results, including fault causes, all probable fault locations and fault maintenance methods, can be deduced to users.

## 7. System Implementation and Validation

### 7.1. System Implementation

**Figure 8:**
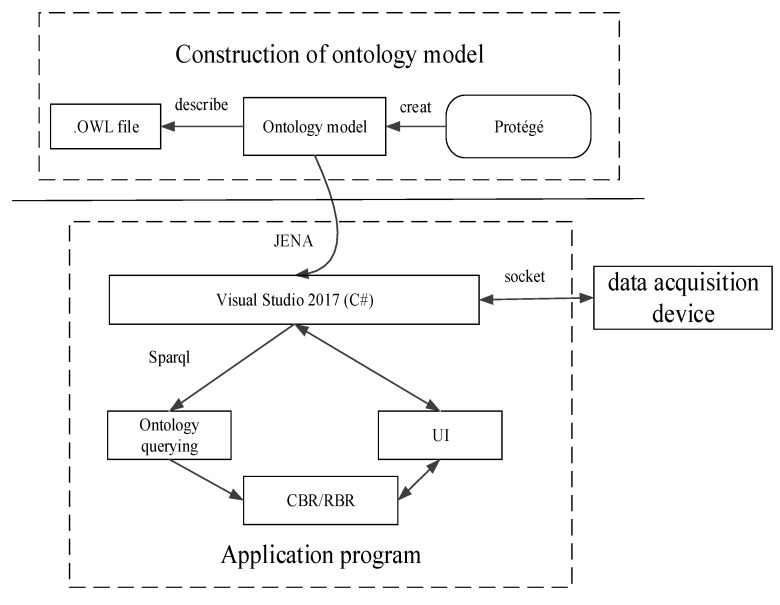
System implementation method for the fault diagnosis of loaders.

This section, describes the implementation of the loaders fault diagnosis system, in accordance with the aforementioned proposed methods. As shown in Figure 8, the system is divided into three parts: the development of the data acquisition device, the construction of the ontology model and the development of the application program. Sensors collect the running parameters, and then data is collected and packed by the data acquisition device. At last, data is transmitted to the remote Visual Studio2017 software development platform through the 3G communication module. The data collected by sensors is of great significance. In the CBR process, the collected data is used to calculate Equation (2). In the RBR module, the collected data is compared with data threshold values to judge instances of the *Parameters* class.

The fault diagnosis ontology model is instantiated by the actual loaders and collected parameters, and then it is built by Protégé software and described by OWL language.

The application program is developed by the Visual Studio2017 software platform and C# language under the Windows 7 system. The socket communication and multithreading technology are used to receive data from the data acquisition device. Jena packages and Java packages are imported into the application program, and then the Jena engineering is used for parsingthe fault diagnosis ontology. The SELECT mode of Sparql is responsible for querying the ontology model to read all properties. CBR and RBR are carried out according to the fifth and sixth chapters in this paper. So far, the loaders fault diagnosis system has been developed.

### 7.2. System Validation

**Figure 9:**
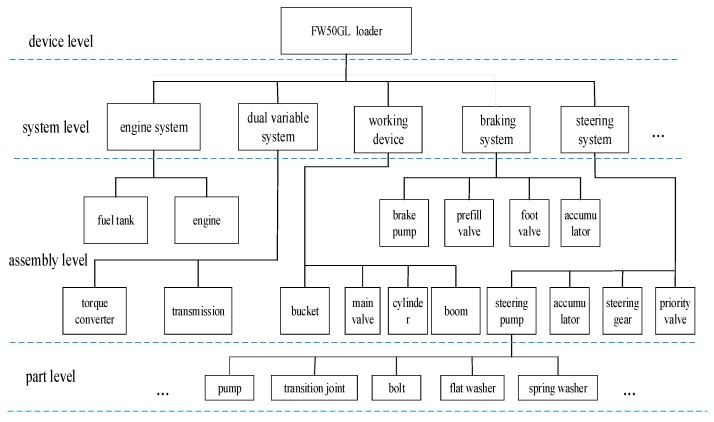
Components of the FW50GL wheel loader.

This section uses the FW50GL wheel loader as an example to verify the validation of the system. As shown in Figure 9, the FW50GL wheel loader is divided into actual components to instantiate the device, system, assembly and part four subclasses in the ontology model, based on layered method mentioned in Section 4.1. Since there are so many components, we mainly describe the major components of the loaders.

**Figure 10:**
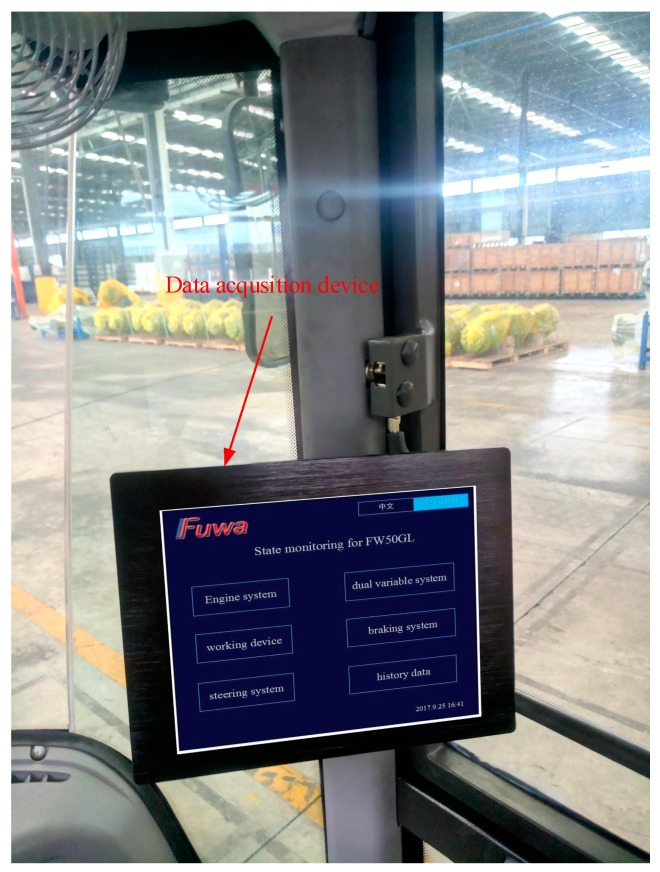
Data acquisition device installed in the cab of the FW50GL wheel loader.

Firstly, as shown in Figure 10, the data acquisition device installed in the cab of the FW50GL wheel loader mainly encapsulates the operating data collected by the sensors. As shown in Table 4, the operating parameters’ names and threshold values of their states are translated into the ontology instances and their data properties, *hasMaxValue* and *hasMinValue*. Every parameter has three instances, for example, instances of engine oil temperature include *engine_oil_temperature_lower_state*, *engine_oil_temperature_noraml_state*, and *engine_oil_temperature_higher_state*. Three instances of each parameter are judged by comparing the data collected by sensors with data threshold values shown in Table 4. For example, when the engine oil temperature collected by a temperature sensor is 60 °C, then the instance of the class, *engine oil temperature*, is *engine_oil_temperature_normal_state,* according to the data threshold values (normal state: 40–120 °C) shown in Table 4.

**Figure 11:**
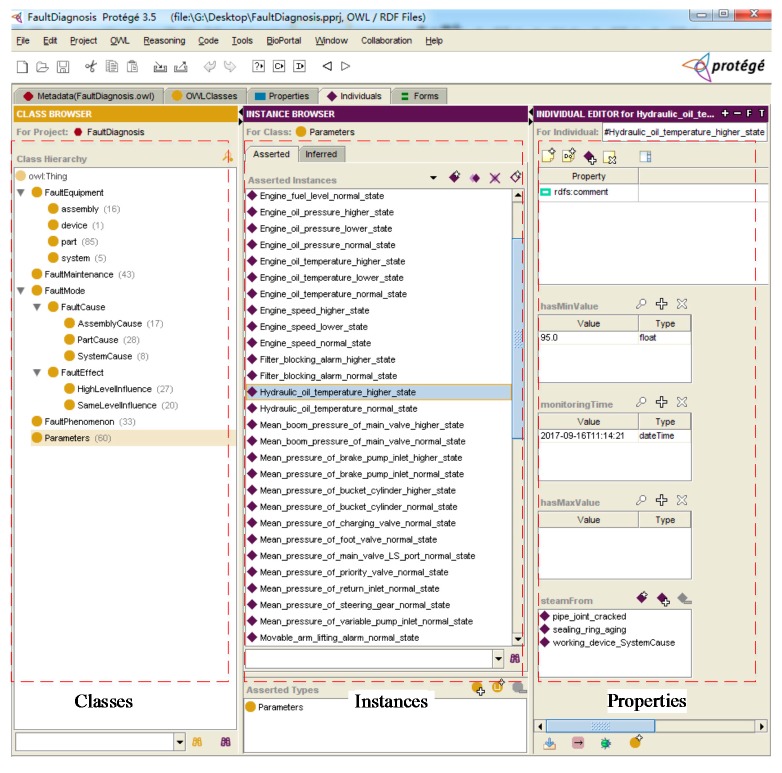
Fault diagnosis ontology model in the Protégé software.

Secondly, Protégé software is used to build the fault diagnosis ontology model, as shown in Figure 11. Classes, instances and properties are listed in order from left to right in Figure 11. Thirdly, the data merging layer is constructed through integrating collected operating parameters data, the built fault diagnosis ontology model, the fault diagnosis rule base and the fault diagnosis case base.

**Figure 12:**
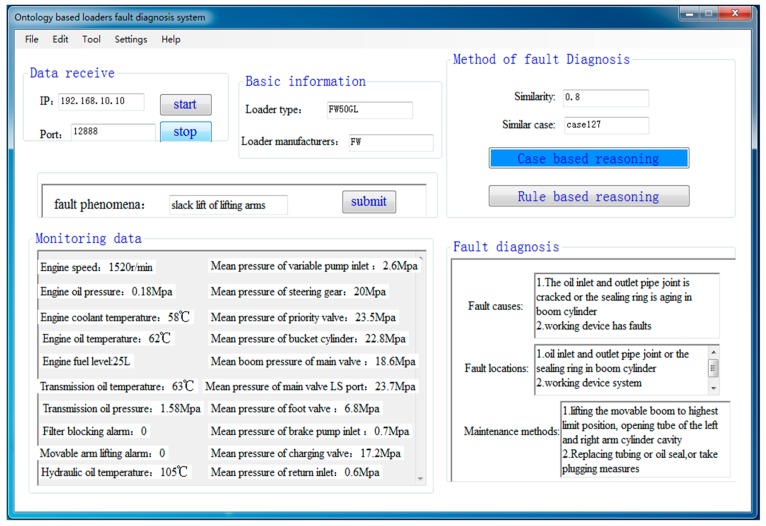
CBR loaders fault diagnosis results.

**Figure 13:**
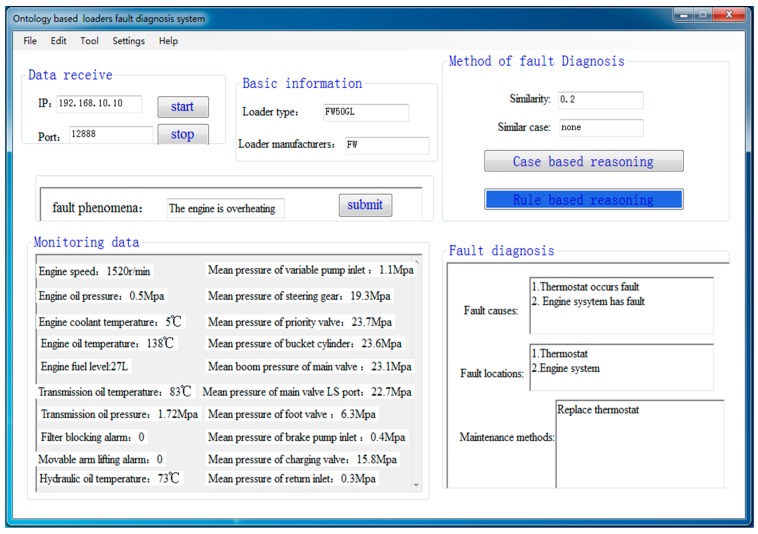
RBR loaders fault diagnosis results.

At last, an application program is developed in this paper to verify the CBR and RBR loaders fault diagnosis methods. The CBR loaders fault diagnosis results are shown in Figure 12. The similarity of case matching is calculated to be 0.8 according to the fault phenomena, loader type and parameters’ feature indexes. This shows that the case matching is successful, and the fault diagnosis results of the matched case are displayed in the application program directly. The RBR loaders fault diagnosis results are shown in Figure 13. This shows that there is no suitable case in the case base when the similarity of case matching is 0.2. The RBR method is carried out to diagnose faults based on defined SWRL rules. Compared with actual failures and diagnostic results in the application program, it is demonstrated that the ontology-based loaders fault diagnosis method is reasonable and effective, and the developed fault diagnosis system can operate correctly.

## 8. Conclusions and Future Work

This paper proposes an ontology-based method for the fault diagnosis of loaders. In this method, an ontology-based fault diagnosis model was introduced to achieve the integrating, sharing and reusing of fault diagnosis knowledge for loaders. Through the integration of CBR and RBR, a universal approach for effective and accurate fault diagnosis of all loaders was realized, with diagnostic results that can be displayed to users more directly in the developed application program. In addition, the program was validated through analyzing a case study.

In particular, in this highly expansible ontology model, the classes, properties and individuals in the fault diagnosis ontology model can be further updated and enhanced. Moreover, the fault diagnosis case base and fault diagnosis rule base are able to be improved and increased continually. Therefore, the fault diagnosis method presented in this paper is highly expansible, which gives it great potential to be applied to all construction machineries and even, hopefully, extended to decision analysis, automatic disassembly, intelligent early warning and other fields.

However, there are still some drawbacks in this method which need to be further strengthened in the future.

(1) The ontology model represented in this paper is relatively simple because the study of loaders fault diagnosis is not very comprehensive. In the future, more classes, object properties, data properties and individuals need to be designed to reflect the complex relationship between fault mode, fault causes and fault effects.

(2) By expanding the rule base and case base by defining more detailed rules and accumulating more cases, more fault diagnosis results (such as recommendation of fault maintenance shops, severity of fault harmfulness, fault alarming level, etc.) will be deduced.

(3) The values $w_{j}$ and $m_{j}$ will be calculated reasonably and accurately based on mathematical methods (such as analytic hierarchy process, expert judgment and fuzzy analysis, etc.) in the future.

(4) In the next step, more actual faults will be accumulated, and several indexes of fault diagnosis performance will be constructed to evaluate the accuracy of the method.

(5) The proposed fault diagnosis method is invalid when the data acquisition device of loaders cannot collect or loses running parameters. Therefore, the probability model of fault locations and fault causes of loaders will be evaluated and diagnosed based on historical operating parameters and the prediction algorithm in the future.

(6) Relative to the correction maintenance mode used in this paper, predictive fault diagnosis and maintenance is more important, because it can repair the faults in advance, so as to avoid greater damage to the loaders caused by faults. To realize predictive maintenance, firstly, auto-regressive and a moving average model and an artificial neural network need to be introduced, to predict operating parameters based on historical operating parameters. Secondly, based on predictive operating parameters, the fault diagnosis method combining ontology, CBR and RBR will still be executed, as in this paper. Finally, predictive fault causes, fault locations and maintenance measures can be achieved.

(7) Although we validated the approach to some extent, it really needs to be empirically evaluated as well. Hence, in the future, we will invite experts and maintenance shop professionals in the loaders fault diagnosis domain to diagnose faults empirically. By combining the approach results and their empirical results, the approach proposed in this paper can be further validated.

## Figures and Tables

**Table 1:** Object properties in the fault diagnosis ontology.

No	Object Property	Domains	Ranges	Inverse Property
1	*stemFrom*	*FaultMode*	*Parameters*	*leadTo*
2	*causeIs*	*FaultMode*	*FaultMode*	N/A
3	*belongTo*	*FaultEquipment*	*FaultEquipment*	N/A
4	*toEffect*	*FaultMode*	*FaultMode*	N/A
5	*toSameLevelEffect*	*FaultMode*	*FaultMode*	N/A
6	*toHighLevelEffect*	*FaultMode*	*FaultMode*	N/A
7	*offerInformation*	*FaultEquipment*	*FaultMaintenance*	N/A
8	*toMaintenance*	*FaultMaintenance*	*FaultMode*	*maintenanceBy*
9	*happenAt*	*FaultMode*	*FaultEquipment*	*hasFault*
10	*hasPhenomenon*	*FaultMode*	*FaultPhenomenon*	*originateFrom*

**Table 2:** Data properties in the fault diagnosis ontology.

No	Data Property	Domains	Ranges	Description
1	*hasNumber*	*FaultEquipment*	string	To record types of components
2	*madeFactory*	*FaultEquipment*	string	To indicate the manufacturer that produces the components
3	*madeTime*	*FaultEquipment*	datetime	To indicate the production time of components
4	*hasMaxValue*	*Parameters*	float	To indicate the maximum value in the normal range of parameters
5	*hasMinValue*	*Parameters*	float	To indicate the minimum value in the normal range of parameters
6	*monitoringTime*	*Parameters*	datetime	To record the monitoring time of parameters
7	*memoryValue*	*FaultMode*	float	To record the memory value of a fault in case library, also used for case-updating

**Table 3:** Fault diagnosis rules.

No	Rules
Rule 1	*FaultMode*(?x) ^*FaultPhenomenon*(?y) ^ *hasPhenomenon*(?x, ?y) → *FaultEquipment*(?z) ^ *happenAt*(?x,?z)
Rule 2	*FaultMode*(?x) ^ *Parameters*(?y) ^ *stemFrom*(?x, ?y) ^ *Device*(?a) ^ *happenAt*(?x, ?a) ^ *FaultMode*(?z) ^ *toHighLevelEffect*(?z, ?x) → *causeIs*(?x, ?z) ^ *SystemCause*(?x)
Rule 3	*FaultMode*(?x) ^ *Parameters*(?y) ^ *stemFrom*(?x, ?y) ^ *System*(?a) ^ *happenAt*(?x, ?a) ^ *FaultMode*(?z) ^ *toHighLevelEffect*(?z, ?x) → *causeIs*(?x,?z) ^ *AsseamblyCause*(?x)
Rule 4	*FaultMode*(?x) ^ *Parameters*(?y) ^ *stemFrom*(?x, ?y) ^*Asseambly*(?a) ^ *happenAt*(?x, ?a) ^ *FaultMode*(?z) ^ *toHighLevelEffect*(?z, ?x) → *causeIs*(?x, ?z) ^ *PartCause*(?x)
Rule 5	*FaultMode*(?x) ^ *Parameters*(?y) ^ *stemFrom*(?x, ?y) ^ *System*(?a) ^ *happenAt*(?x, ?a) ^ *FaultMode*(?z) ^ *toSameLevelEffect*(?z, ?x) → *causeIs*(?x, ?z) ^ *SystemCause*(?x)
Rule 6	*FaultMode*(?x) ^ *Parameters*(?y) ^ *stemFrom*(?x, ?y) ^ *Asseambly* (?a) ^ *happenAt*(?x, ?a) ^ *FaultMode*(?z) ^ *toSameLevelEffect*(?z, ?x) → *causeIs*(?x, ?z) ^ *Asseambly Cause*(?x)
Rule 7	*FaultMode*(?x) ^ *Parameters*(?y) ^ *stemFrom*(?x, ?y) ^*Part*(?a) ^ *happenAt*(?x, ?a) ^ *FaultMode*(?z) ^ *toSameLevelEffect*(?z, ?x) → *causeIs*(?x, ?z) ^ *PartCause*(?x)
Rule 8	*FaultMode*(?x) ^ *FaultEquipment*(?y) ^ *happenA*t(?x,?y) ^ *PartCause*(?x) → *Part*(?y)
Rule 9	*FaultMode*(?x) ^ *FaultEquipment*(?y) *happenAt* (?x,?y) ^ *AsseamblyCause*(?x) → *Asseambly*(?y)
Rule 10	*FaultMode*(?x) ^ *FaultEquipment*(?y) ^ *happenAt*(?x,?y) ^ *SystemCause*(?x) → *System*(?y)
Rule 11	*FaultMode*(?x) ^ *FaultEquipment*(?y) ^ *happenAt*(?x,?y) ^ *FaultMaintenance*(?z) ^ *offInformation*(?y,?z) → *toMaintenance*(?z, ?x)

**Table 4:** Operating parameters and threshold values of their states.

No	Parameters	Abnormal State 1 below Normal Value	Normal State	Abnormal State 2 above Normal Value
1	Engine speed	≤680 r/min	680–2400 r/min	≥2400 r/min
2	Engine oil pressure	≤0.07 Mpa	0.07–0.4 Mpa	≥0.4 Mpa
3	Engine coolant temperature	≤0 °C	0–110 °C	≥110 °C
4	Engine oil temperature	≤40 °C	40–120 °C	≥120 °C
5	Engine fuel level	≤6.25 L	6.25–50 L	≥50 L
6	Transmission oil temperature	≤0 °C	0–127 °C	≥127 °C
7	Transmission oil pressure	≤0.36 Mpa	0.36–2.24 Mpa	≥2.24 Mpa
8	Filter blocking alarm	>0	≤0 (0:normal 1:alarming)	>0
9	Movable arm lifting alarm	>0	≤0 (0:normal 1:alarming)	>0
10	Hydraulic oil temperature	≤0 °C	0–95 °C	≥95 °C
11	Mean pressure of variable pump inlet	≤0 Mpa	0–3 Mpa	≥3 Mpa
12	Mean pressure of steering gear	≤18 Mpa	18–21 Mpa	≥21 Mpa
13	Mean pressure of priority valve	≤22 Mpa	22–24 Mpa	≥24 Mpa
14	Mean pressure of bucket cylinder	≤22 Mpa	22–24 Mpa	≥24 Mpa
15	Mean boom pressure of main valve	≤22 Mpa	22–24 Mpa	≥24 Mpa
16	Mean pressure of main valve LS port	≤22 Mpa	22–24 Mpa	≥24 Mpa
17	Mean pressure of foot valve	≤6 Mpa	6–7 Mpa	≥7 Mpa
18	Mean pressure of brake pump inlet	≤0 Mpa	0–1 Mpa	≥1 Mpa
19	Mean pressure of charging valve	≤15 Mpa	15–18 Mpa	≥18 Mpa
20	Mean pressure of return inlet	≤0 Mpa	0–1 Mpa	≥1 Mpa
